# Uncontrolled hypertension is associated with increased risk of graft failure in kidney transplant recipients: a nationwide population-based study

**DOI:** 10.3389/fcvm.2023.1185001

**Published:** 2023-07-14

**Authors:** Chang Seong Kim, Tae Ryom Oh, Sang Heon Suh, Hong Sang Choi, Eun Hui Bae, Seong Kwon Ma, Jin Hyung Jung, Bongseong Kim, Kyung-Do Han, Soo Wan Kim

**Affiliations:** ^1^ Department of Internal Medicine, Chonnam National University Medical School, Gwangju, Republic of Korea; ^2^ Department of Internal Medicine, Chonnam National University Hospital, Gwangju, Republic of Korea; ^3^ Department of Biostatistics, College of Medicine, Catholic University of Korea, Seoul, Republic of Korea; ^4^ Department of Statistics and Actuarial Science, Soongsil University, Seoul, Republic of Korea

**Keywords:** kidney, transplantation, hypertension, graft failure, risk

## Abstract

**Backgroud:**

Hypertension is highly prevalent in patients with kidney transplantation caused by transplantation-related immunologic or non-immunologic risk factors. However, whether a strict definition of hypertension (≥130/80 mmHg) and subdivided blood pressure (BP) groups are associated with an increased risk of graft failure after kidney transplantation using a nationwide large cohort study are still unknown.

**Methods:**

Using Korean National Health Insurance Service data, we included 14,249 patients who underwent kidney transplantation from 2002 to 2016. Patients were categorized into five BP groups according to the 2021 Kidney Disease: Improving Global Outcomes practice guidelines for BP management: normal BP (<120/80 mmHg), elevated BP (120–129/ < 80 mmHg), incident hypertension (≥130/80 mmHg), and controlled or uncontrolled hypertension with anti-hypertensive medications.

**Results:**

The primary outcome was graft failure, which occurred in 1934 (13.6%) participants during the 6-year follow-up. After adjusting for covariates, hypertension was associated with a higher risk of graft failure [Adjusted hazard ratio (AHR), 1.70; 95% confidence interval (CI), 1.48–1.96)] than no-hypertension. The AHR for graft failure was the highest in patients with uncontrolled hypertension (AHR, 2.13; 95% CI, 1.80–2.52). The risk of graft failure had a linear relationship with systolic and diastolic BP, and pulse pressure.

**Conclusions:**

In this nationwide population-based study, hypertension ≥130/80 mmHg based on the 2021 KDIGO BP guidelines in kidney transplantion recipients, and elevated systolic and diastolic BP, and pulse pressure were associated with the risk of developing graft failure in kidney transplant recipients.

## Introduction

Hypertension is highly prevalent in patients with kidney transplantation (1–3). Various factors affect blood pressure (BP) in kidney transplant recipients (KTR) including acute and chronic renal allograft dysfunction, retained native kidney, denervated transplanted kidney, and the regular use of calcineurin inhibitors and steroids (3–5). These factors may impair the autoregulation of BP or result in sodium and water retention (6, 7). After kidney transplantation, increased blood pressure is associated with deleterious allograft and patient survival (8–13). Therefore, optimal BP management is essential to improve graft outcomes and mortality rates.

Recently, the target of BP management was lowered to <120 mmHg in patients with chronic kidney disease based on the Systolic BP Intervention Trial (SPRINT), in which the intensive lowering of clinic systolic BP (SBP) reduced the risk for cardiovascular disease and all-cause mortality (14). On the other hand, the 2021 Kidney Disease: Improving Global Outcomes (KDIGO) practice guidelines for BP management in adult KTR still recommends a target of <130/80 mmHg using standardized office BP measurement, consistent with the previous 2009 KDIGO BP guidelines for KTR (15, 16). Nevertheless, previous studies did not give a confirmative result to an increased risk of graft failure when the target kidney transplant recipient BP was ≥130/80 mmHg because they adopted an old definition of hypertension (8–11, 13, 17). Therefore, in this large nationwide population-based study, we investigated the association between hypertension based on the definition of 2021 KDIGO guidelines for KTR, subdivided BP components, and the risk of graft failure among patients with kidney transplants.

## Materials and methods

### Korean national health insurance service (KNHIS) data

In this study, we used a national health insurance claims database established by the KNHIS, which includes all claims data provided by the KNHIS and Medical Aid programs. Data extracted from the KNHIS database were considered representative of the entire South Korean population, and the details of this database have been previously described (18). Depending on their occupations, all insured Koreans undergo an annual or biennial health examination that is supported by the KNHIS. Anonymized data are publicly available from the National Health Insurance Sharing Service and can be accessed at https://nhiss.nhis.or.kr/bd/ab/bdaba000eng.do. The study protocol was approved by the Institutional Review Board of Chonnam National University Hospital (CNUH-EXP-2022-274) and has therefore been performed in accordance with the ethical standards laid down in the 1964 Declaration of Helsinki and its later amendments. The requirement for written informed consent was waived by the review board because anonymous and de-identified information was used for analysis.

### Main study population and follow-up

**Figure 1:**
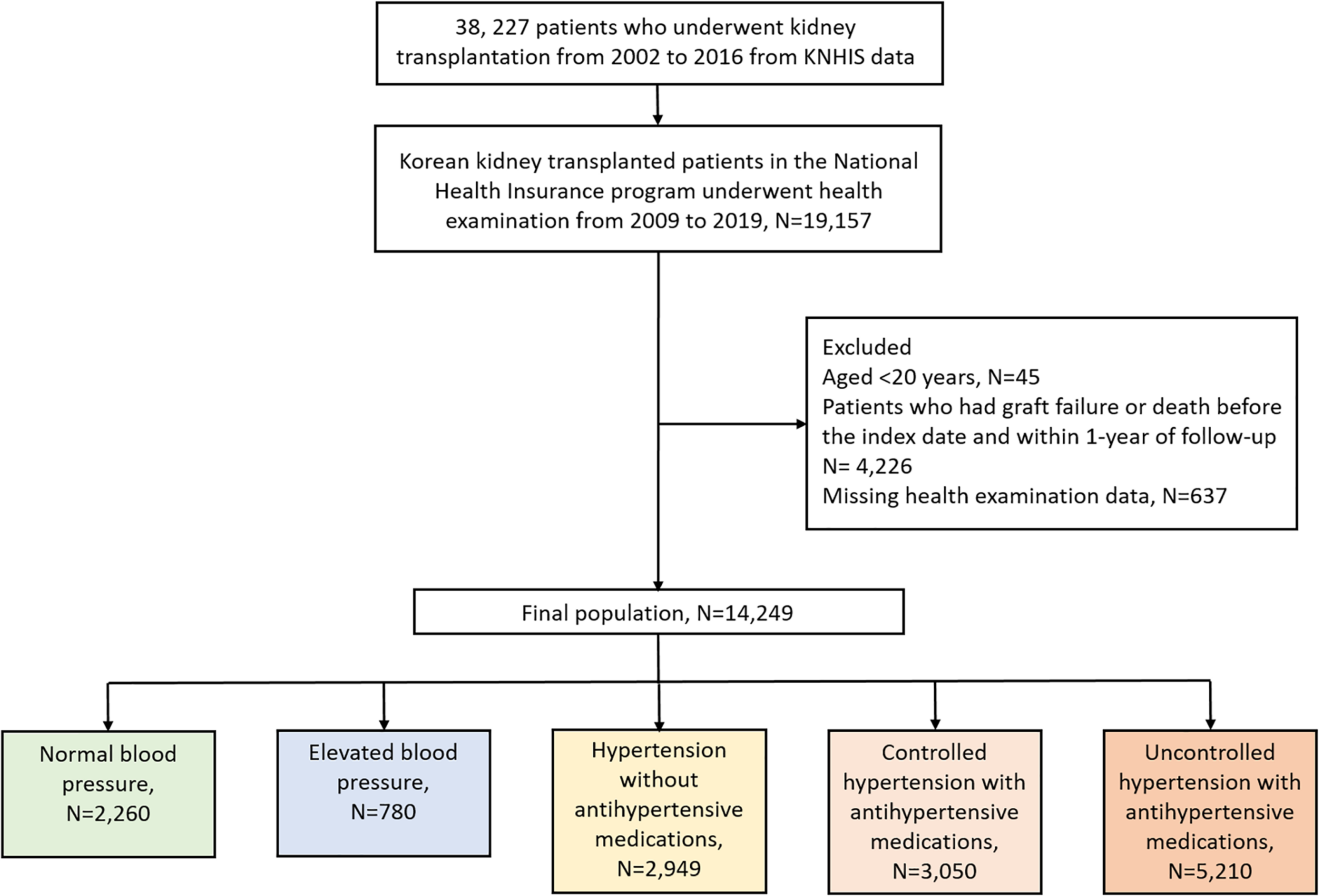
Flowchart of participant enrollment.

Initially, 38, 227 patients who underwent kidney transplantation from 2002 to 2016 were identified. Of these, we included patients who had undergone health checkups from 2009 to 2017 because the questionnaire form changed in 2009. The index date was the date of the first health check-up after 2009. We excluded those aged <20 years, and those with graft failure or death before the index date and within 1-year of follow-up. We also excluded subjects with missing health examination data. Finally, 14, 249 KTR were included in the study and were followed-up from the index date to the date of graft failure during the follow-up period, death, loss of health insurance qualification, or the end of the study period (December 31, 2019). A detailed enrollment flowchart is shown in Figure 1.

### Definitions

BP was measured by trained clinicians at least twice, using a mercury or automatic sphygmomanometer with the participants in a sitting position following a minimum of 5 min of rest in the index date. Hypertension was defined as SBP ≥ 130 mmHg or diastolic BP (DBP) ≥ 80 mmHg in the health examination database or a history of using antihypertensive medications according to the 2021 KDIGO BP guidelines and 2017 American College of Cardiology/American Heart Association guidelines (15, 19). Moreover, participants were classified into five hypertension groups as follows: (1) normal BP (<120/80 mmHg, patients with no prior diagnosis of hypertension); (2) elevated BP (120–129/ < 80 mmHg, but those with no prior diagnosis of hypertension); (3) incident hypertension without medication (≥130/80 mmHg, but not taking antihypertensive medications; (4) controlled hypertension (<130/80 mmHg, patients diagnosed with and taking medication for hypertension); and (5) uncontrolled hypertension (≥130/80 mmHg, patients diagnosed with and taking medication for hypertension). Participants were also classified into five groups based on their measured SBP: (1) <100 mmHg; (2) 100–119 mmHg; (3) 120–129 mmHg; (4) 130–139 mmHg; (5) ≥140 mmHg for SBP; DBP (1) <70 mmHg; (2) 70–79 mmHg; (3) 80–89 mmHg; (4) 90–99 mmHg; (5) ≥100 mmHg, as well as pulse pressure (PP) defined by SBP minus DBP (1) <40 mmHg; (2) 40–49 mmHg; (3) 50–59 mmHg; (4) 60–69 mmHg; (5) ≥70 mmHg. For each participant, the body mass index was calculated by dividing the body weight (kg) by the height squared (m^2^). We defined obesity as a body mass index ≥ 25 kg/m^2^ according to the WHO recommendations for Asian populations (20). The estimated glomerular filtration rate (eGFR) was calculated using the Modification of Diet in Renal Disease formula. Data on age, sex, health behaviour-related factors, and other definitions of smoking status, alcohol consumption, regular exercise, and diagnosis for diabetes, cardiovascular disease and dyslipidemia are described in Supplementary Table S1.

### Study outcomes

The study outcome was incident death-censored graft failure, defined as the presence of hemodialysis, peritoneal dialysis, or kidney re-transplantation. Patients with death-censored graft failure were identified using a combination of International Classification of Diseases Tenth Revision, Clinical Modification (ICD-10-CM) codes (N18–19, Z49, Z94.0, and Z99.2) and a special code (V001, procedure-related outpatient care or inpatient treatment on the day of hemodialysis; V003, peritoneal dialysis) at least three times during 3 months, and kidney transplantation code (V005). We excluded patients with a dialysis code on the same date as an acute kidney failure code (N17.9). In the event of death with a functioning graft, the follow-up period was censored at the date of death.

### Statistical analyses

Continuous variables are described as the mean ± standard deviation and categorical variables are presented as numbers with proportions. Intergroup differences were tested using the chi-squared test or Student's *t*-test, as appropriate. The incidence rates of graft failure are presented as the number of cases calculated per 1,000 person-years. The cumulative incidence probability of graft failure was estimated using the Kaplan–Meier method, and between-group comparisons of the resulting curves were subjected to univariate analysis via the log-rank test. Multivariable analyses were performed using Cox proportional hazard regression models, and calculated hazard ratios (HRs) with 95% confidence intervals (CIs). The proportional hazards assumption was tested visually with the Schoenfeld residual plots. Model 1 was adjusted for age and sex. Model 2 was adjusted for age, sex, income level, smoking status, alcohol consumption status, physical activity, eGFR, obesity, diabetes mellitus, cardiovascular disease and dyslipidemia. Model 3 included all covariates in Model 2, along with the use of antihypertensive medications (diuretics, calcium channel blockers, β-blockers, α-blocker, angiotensin converting enzyme inhibitors, and/or angiotensin receptor blockers). Smooth cubic spline HR curves for the graft failure were plotted after adjusting for all covariates (Model 3). Subgroup analyses were conducted according to age, sex, smoking status, and diabetes as well as the duration from kidney transplantation to BP measurement. Interaction terms were added to test for effect modification across the subgroups. All statistical analyses were performed using the Statistical Analysis System (SAS) software (version 9.4; SAS Institute, Cary, NC, USA). All significance tests were 2-tailed and *P-*values* *< 0.05 were considered statistically significant.

## Results

### Baseline characteristics

The mean baseline age of the participants was 50.9 years, and 58.2% were men. The baseline characteristics of the study population according to hypertension are presented in Table 1. Of the total population, 11,209 (78.7%) KTR were diagnosed with hypertension. Among those with hypertension, 2949 (26.3%), 3050 (27.2%), and 5210 (46.5%) participants had incident hypertension, controlled, and uncontrolled hypertension, respectively. Participants with hypertension were more likely than those without hypertension to be male, older, smokers, take regular exercise, obese, and with a higher prevalence of diabetes, cardiovacular disease and dyslipidemia than those without hypertension.

**Table 1:** Baseline characteristics of the study population.

Characteristics	Total	No hypertension	Hypertension	*P* value
Number of patients (%)	14,249 (100)	3,040 (21.3)	11,209 (78.7)	
Age, mean ± SD, years	50.9 ± 10.9	49.0 ± 11.0	51.4 ± 10.9	<0.001
20–39	1,956 (13.7)	507 (16.7)	1,449 (12.9)	<0.001
40–64	10,885 (76.4)	2,323 (76.4)	8,562 (76.4)	
≥65	1,408 (9.9)	210 (6.9)	1,198 (10.7)	
Sex, male (%)	8,299 (58.2)	1,302 (42.8)	6,997 (62.4)	<0.001
Smoking (%)				
Never	8,997 (63.1)	2,153 (70.8)	6,844 (61.1)	
Former	3,851 (27.0)	614 (20.2)	3,237 (28.9)	<0.001
Current	1,401 (9.8)	273 (9.0)	1,128 (10.1)	
Five hypertension groups (%)				
Normal BP	2,260 (15.9)	2,260 (74.3)		
Elevated BP	780 (5.5)	780 (25.7)		
Incident hypertension without medication	2,949 (20.7)		2,949 (26.3)	
Controlled hypertension	3,050 (21.4)		3,050 (27.2)	
Uncontrolled hypertension	5,210 (36.6)		5,210 (46.5)	
Alcohol consumption (%)	2,905 (20.4)	621 (20.4)	2,284 (20.4)	0.951
Regular physical activity (%)	3,181 (22.3)	629 (20.7)	2,552 (22.8)	0.015
Low income (%)	3,911 (27.5)	835 (27.5)	3,076 (27.4)	0.978
Diabetes mellitus (%)	4,259 (29.9)	617 (20.3)	3,642 (32.5)	<0.001
CVD (%)	613 (4.3)	79 (2.6)	534 (4.8)	<0.001
Dyslipidemia (%)	7,380 (51.8)	1,128 (37.1)	6,252 (55.8)	<0.001
WC, mean ± SD, cm	80.3 ± 9.5	76.9 ± 9.0	81.2 ± 9.4	<0.001
Height, mean ± SD, cm	164.0 ± 8.7	162.4 ± 8.5	164.4 ± 8.7	<0.001
Weight, mean ± SD, cm	62.2 ± 11.2	58.5 ± 10.2	63.2 ± 11.3	<0.001
BMI, mean ± SD, kg/m^2^	23.1 ± 3.3	22.1 ± 3.0	23.3 ± 3.3	<0.001
Obesity (BMI ≥ 25 kg/m^2^)	3,661 (25.7)	514 (16.9)	3,147 (28.2)	<0.001
Fasting glucose, mean ± SD, mg/dl	105.2 ± 33.3	100.6 ± 28.9	106.4 ± 34.3	<0.001
Total cholesterol, mean ± SD, mg/dl	184.2 ± 37.3	182.6 ± 35.7	184.7 ± 37.7	0.007
Antihypertensive medications^a^				
Diuretics (%)	2,057 (14.4)		2,057 (14.4)	
Calcium channel blockers (%)	5,795 (40.7)		5,795 (40.7)	
*β*-blockers (%)	1,702 (11.9)		1,702 (11.9)	
Angiotensin converting enzyme inhibitors (%)	683 (4.8)		683 (4.8)	
Angiotensin receptor blockers (%)	3,849 (27.0)		3,849 (27.0)	
Follow-up duration, mean ± SD, years	6.0 ± 2.9	6.3 ± 2.7	5.9 ± 2.9	<0.001

BP, blood pressure; HTN, hypertension; BMI, body mass index; CVD, cardiovascular disease; WC, waist circumference; SD, standard deviation.

^a^ Claim within 1 year from index date.

### Hypertension and risk of graft failure

**Figure 2:**
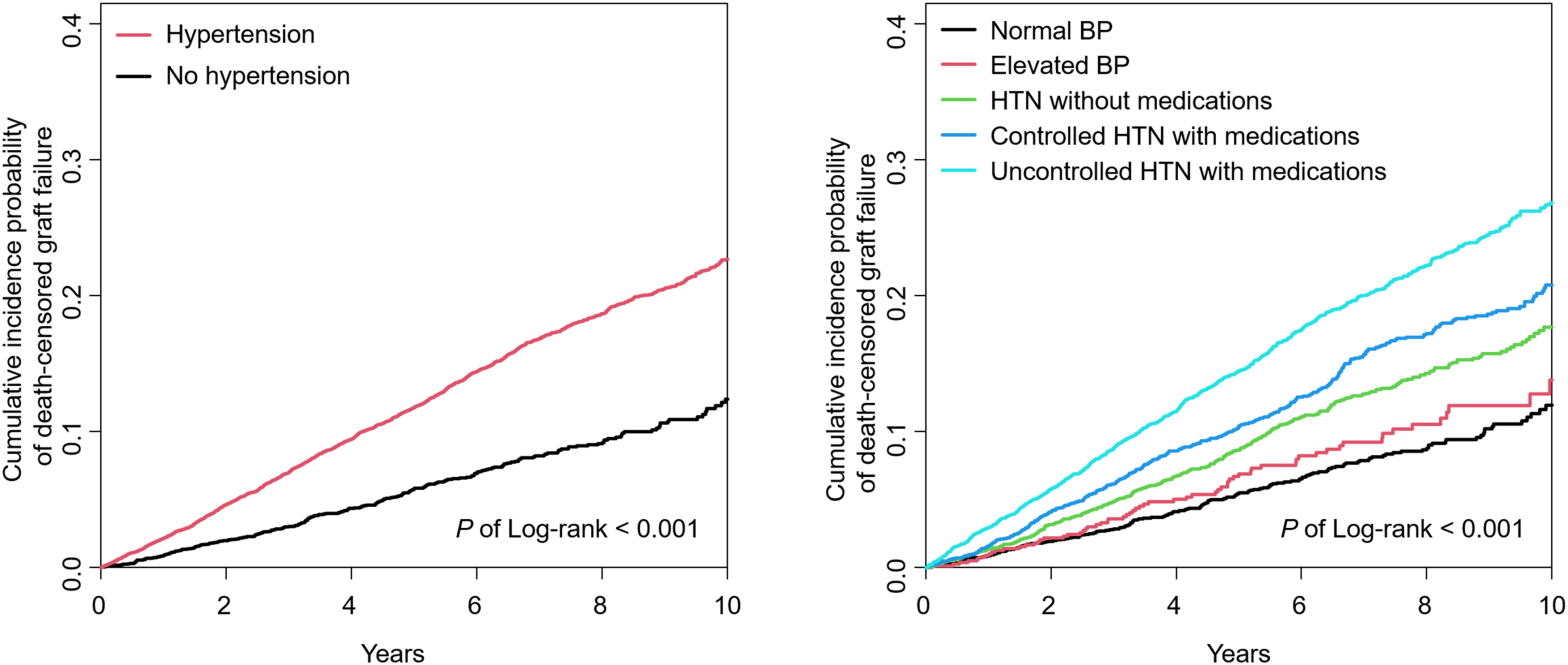
Kaplan–Meier curve for the incidence probability of death-censored graft failure with or without hypertension, and five hypertension groups. BP, blood pressure; HTN, hypertension.

During a mean follow-up period of 6.0 ± 2.9 years, 1934 (13.6%) participants developed graft failure. The incidence rates of graft failure were 12.2 and 25.3 (per 1,000 person-years) in patients without and with hypertension, respectively. The incidence rates of graft failure according to hypertension groups were 11.7, 13.8, 18.9, 22.8, and 31.3 for normal BP, elevated BP, incident hypertension, and controlled and uncontrolled hypertension with antihypertensive medications, respectively (Table 2). After adjusting for confounding factors (Cox Model 2), hypertensive patients had a significantly higher risk of graft failure than those without hypertension (adjusted HR, 1.703; 95% CI, 1.482–1.957). In the five hypertension groups, adjusted HRs for each group were 1 (reference), 1.198, 1.461, 1.590, and 2.127, respectively. Uncontrolled hypertension in the antihypertensive group had the highest risk for graft failure (adjusted HR, 2.127; 95% CI, 1.799–2.515). Kaplan-Meier curves for the incidence probability of graft failure according to hypertension and the five groups are shown in Figure 2, and similar results were obtained.

**Table 2:** Incidence rates and HRs of death-censored graft failure according to hypertension categories.

Group	Number of participants	Graft failure	Follow-up Duration, Person-years	Incidence Rate, Per 1,000 person-years	Unadjusted, HR (95% CI)	Model 1, HR (95% CI)^a^	Model 2, HR (95% CI)^b^
Hypertension							
No	3,040	235	19,257	12.2	1 (reference)	1 (reference)	1 (reference)
Yes	11,209	1,699	66,545	25.5	2.099 (1.831–2.406)	2.085 (1.816–2.393)	1.702 (1.481–1.956)
Hypertension categories							
Normal BP	2,260	170	14,537	11.7	1 (reference)	1 (reference)	1 (reference)
Elevated BP	780	65	4,720	13.8	1.180 (0.886–1.570)	1.190 (0.894–1.584)	1.199 (0.900–1.596)
Incident HTN without medications	2,949	349	18,431	18.9	1.622 (1.351–1.949)	1.625 (1.351–1.953)	1.464 (1.217–1.760)
Controlled HTN	3,050	418	18,300	22.8	1.960 (1.640–2.342)	1.969 (1.645–2.358)	1.589 (1.326–1.905)
Uncontrolled HTN	5,210	932	29,815	31.3	2.687 (2.282–3.165)	2.708 (2.293–3.197)	2.125 (1.797–2.513)

BP, blood pressure; HR, hazard ratio; CI, confidential interval; SBP, systolic blood pressure; DBP, diastolic blood pressure; HTN, hypertension.

^a^ Model 1 was adjusted for age and sex.

^b^ Model 2 was adjusted for age, sex, low income, smoking, alcohol consumption, regular exercise, obesity, estimated glomerular filtration rate, and history of diabetes, cardiovascular disease and dyslipidemia.

**Figure 3:**
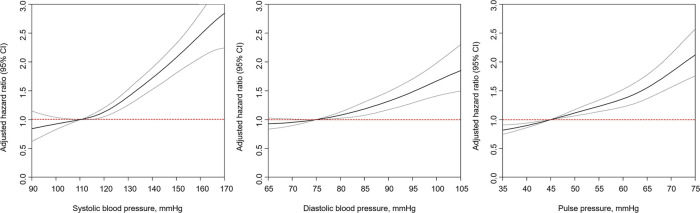
Smoothed hazard ratios curves of the associations of SBP, DBP, and PP with death-censored graft failure in kidney transplant recipients in adjusted Cox-model 3. CI, confidence interval.

Participants were also classified based on SBP, DBP, and PP levels. The incidence rates and adjusted HRs (Cox Model 3) of graft failure were remarkably increased with an increase in the SBP, DBP, and PP in each group compared with the reference group (Table 3). These associations were confirmed by smooth HR curve analyses even after multivariable adjustments (Figure 3).

**Table 3:** Incidence rates and HRs of death-censored graft failure according to blood pressure.

Group	Number of participants	Graft failure	Follow-up Duration, Person-years	Incidence Rate, Per 1,000 person-years	Unadjusted, HR (95% CI)	Model 1, HR (95% CI)^a^	Model 2, HR (95% CI)^b^	Model 3, HR (95% CI)^c^
SBP, mmHg								
<100	394	32	2,432	13.2	0.795 (0.556–1.138)	0.813 (0.568–1.164)	0.763 (0.533–1.093)	0.790 (0.552–1.131)
100–119	4,401	456	27,587	16.5	1 (reference)	1 (reference)	1 (reference)	1 (reference)
120–129	3,705	454	22,495	20.2	1.222 (1.073–1.392)	1.219 (1.070–1.388)	1.173 (1.030–1.336)	1.151 (1.010–1.311)
130–139	3,592	561	21,442	26.2	1.586 (1.402–1.795)	1.587 (1.401–1.797)	1.448 (1.278–1.640)	1.400 (1.235–1.586)
≥140	2,157	431	11,847	36.4	2.212 (1.939–2.524)	2.242 (1.962–2.562)	1.894 (1.657–2.164)	1.796 (1.571–2.055)
DBP, mmHg								
<70	2,391	261	14,370	18.2	0.931 (0.805–1.077)	0.946 (0.817–1.094)	0.935 (0.808–1.082)	0.947 (0.818–1.096)
70–79	4,948	590	30,234	19.5	1 (reference)	1 (reference)	1 (reference)	1 (reference)
80–89	5,120	764	30,893	24.7	1.269 (1.139–1.412)	1.259 (1.130–1.402)	1.207 (1.084–1.345)	1.194 (1.072–1.330)
90–99	1,348	220	7,921	27.8	1.424 (1.220–1.663)	1.417 (1.214–1.655)	1.274 (1.090–1.489)	1.230 (1.052–1.437)
≥100	442	99	2,385	41.5	2.137 (1.727–2.644)	2.103 (1.699–2.602)	1.859 (1.501–2.302)	1.807 (1.459–2.238)
Pulse pressure, mmHg								
<40	2,392	227	14,713	15.4	0.792 (0.681–0.920)	0.795 (0.684–0.924)	0.779 (0.671–0.906)	0.790 (0.680–0.918)
40–49	5,709	691	35,461	19.5	1 (reference)	1 (reference)	1 (reference)	1 (reference)
50–59	4,220	661	24,916	26.5	1.364 (1.226–1.518)	1.380 (1.240–1.537)	1.235 (1.110–1.376)	1.205 (1.082–1.342)
60–69	1,433	235	8,140	28.9	1.487 (1.282–1.724)	1.539 (1.325–1.787)	1.289 (1.109–1.498)	1.241 (1.067–1.442)
≥70	495	120	2,573	46.6	2.411 (1.986–2.926)	2.572 (2.112–3.133)	1.976 (1.620–2.409)	1.890 (1.550–2.306)

BP, blood pressure; HTN, hypertension; HR, hazard ratio; CI, confidential interval; SBP, systolic blood pressure; DBP, diastolic blood pressure.

^a^ Model 1 was adjusted for age and sex.

^b^ Model 2 was adjusted for age, sex, low income, smoking, alcohol consumption, regular exercise, obesity, estimated glomerular filtration rate, and history of diabetes, cardiovascular disease and dyslipidemia.

^c^ Model 3 was adjusted for age, sex, low income, smoking, alcohol consumption, regular exercise, obesity, estimated glomerular filtration rate, and history of diabetes, cardiovascular disease and dyslipidemia, use of antihypertensive medications.

### Subgroup analyses

**Figure 4:**
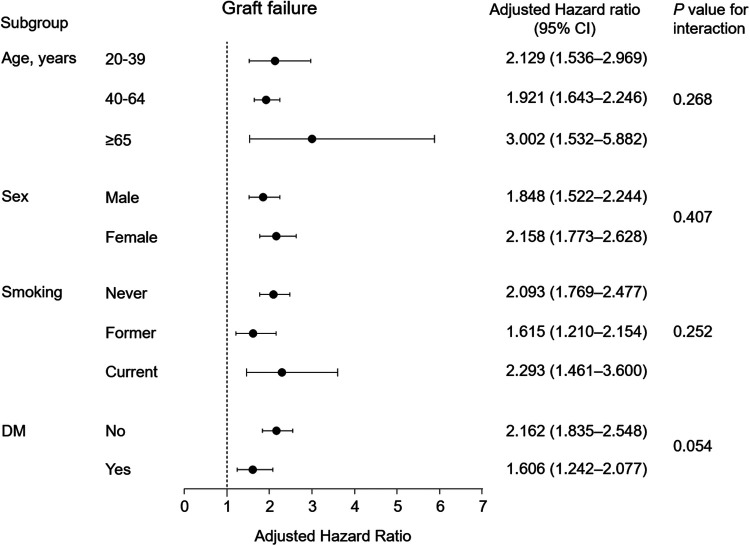
Subgroup analysis of the association between hypertension and death-censored graft failure in adjusted Cox-model 3. Points and bars represent hazard ratio estimates and their associated 95% CIs, respectively. CI, confidential interval; DM, diabetes mellitus.

For subgroup analyses according to age, the participants were classified into <40, 40–65, and ≥65 years. In all subgroup analyses according to age, sex, smoking, and diabetes mellitus, hypertension was consistently associated with the risk of graft failure, and there was no significant difference between the subgroups (Figure 4).

We also analyzed the association between the five hypertension groups, the SBP and DBP groups, and PP groups with graft failure among subgroups (Supplementary Tables S2−S5). The adjusted HRs indicated no significant differences between participants regardless of age group or sex. However, the increased risk of graft failure according to advanced hypertension groups and high SBP and DBP was significantly higher in participants without diabetes than in those with diabetes.

To determine the association between the duration from kidney transplantation to BP measurement and graft failure, participants were classified into two groups; <5 years and ≥5 years based on this duration. The results were found to be consistent in both subgroups of participants (Supplementary Table S6).

## Discussion

The present study demonstrated that (1) the presence of hypertension (≥130/80 mmHg) as based on the 2021 KDIGO BP guidelines for KTR increased the risk of graft failure; (2) uncontrolled hypertension (≥130/80 mmHg) albeit taking antihypertensive medications had the highest risk (2.1-fold) of graft failure compared with normal BP; (3) the risk of graft failure increased gradually as the SBP, DBP, and PP increased; (4) this association was present in the ≥120 mmHg SBP, ≥80 mmHg DBP, and ≥50 mmHg of PP groups.

Hypertension after kidney transplantation is common, although the prevalence ranges from 50% to 90% and varies depending on the definition, population, and use of antihypertensive medications (8, 21–24). Sustained hypertension is an established risk factor for worsening kidney function, cardiovascular morbidity, and mortality (25). Therefore, it is important to establish optimal BP control in relation to graft survival in KTR. A previous study of 392 allograft recipients from living donors showed that SBP and DBP levels during the first year after transplantation were associated with renal allograft failure, which was independent of renal function (9). Similarly, in a study of KTR from deceased donors at the same center, a 10 mmHg increment in BP in the first year post-transplantation strongly predicted allograft failure (26). In a large retrospective study using the Collaborative Transplant Study data, 24,404 KTR with an SBP >140 mmHg at 1-year post-transplantation but controlled to ≤140 mmHg at 3 years had improved long-term renal allograft survival compared with those with a persistent SBP of >140 mmHg to 3 years (10). However, these results did not provide a definite cutoff for BP target regarding the risk of graft survival.

Our results are consistent with a previous hypothesis that a higher BP is associated with an increased risk of graft failure. Especially, hypertension of ≥130/80 mmHg, as per the 2021 KDIGO BP guidelines, in KTR increased the risk of death-censored graft failure by 1.7-fold compared with non-hypertension. Furthermore, an elevated SBP or DBP of ≥120 mmHg/80 mmHg had a significant association with graft failure, suggesting that a modestly increased BP in KTR could worsen kidney function. Although a retrospective study of 815 KTR who achieved a mean SBP <130 mmHg showed a lower mortality rate, these results were not maintained in graft survival (12). In addition, a secondary analysis of the Folic Acid for Vascular Outcome Reduction in Transplantation trial of 3,598 KTR, found no associations of SBP or DBP with composite outcomes defined as a decline in ≥50% of eGFR or dialysis (17). Although our findings are not consistent with previous studies, this might be related to the relatively small sample size and different primary endpoints of previous studies (12, 17).

Our results also demonstrated that the risk of graft failure was associated with a linear relationship with SBP, DBP, or PP. Moreover, the HRs of uncontrolled hypertension, even while taking antihypertensive medication, increased the risk 2.1-fold than that of normal BP. Considering the results of our study and previous studies (9, 26), achieving an intensive BP target of <130/80 mmHg might be important for decreasing the risk of graft failure in KTR.

Another important finding of this study was that increased PP had a linear association with the risk of graft failure. It is generally accepted that PP, which reflects arterial stiffness, is linked to the progression of CKD and cardiovascular mortality (27), suggesting it might be a good surrogate marker for predicting graft failure in KTR.

The strength of this study was the enrollment of a large population of approximately 14,000 KTR from a nationwide health checkup database over a relatively long follow-up duration. Because of the large population, we classified the patients into several hypertensive groups and subdivided BP groups to determine the association between a lower hypertension definition and various BP levels and the development of graft failure. This study had several limitations. First, we used a single BP measurement taken in the office to determine hypertension. However, BP variability is common in patients with kidney disease, and office BP measurements do not reflect nocturnal hypertension, masked hypertension, and white coat hypertension (28, 29). Second, we did not capture data on allograft rejection and use of immunosuppressive agents for KTR that could affect allograft failure due to the nature of this retrospective study. Third, there was a possibility of coding inaccuracies due to limitations by an administrative database. Fourth, our findings cannot be generalized to other ethnic groups, because this study was limited to the Korean population. Finally, this was a retrospective study design; therefore, randomized controlled trials to examine optimal BP levels in KTR to prolong graft survival are needed in the future.

In conclusion, this Korean nationwide population-based cohort study found that hypertension ≥130/80 mmHg based on the 2021 KDIGO BP guidelines in KTR, as well as elevated SBP, DBP, and PP were associated with the risk of developing graft failure in patients with kidney transplantation after adjusting for various covariates. Whether intensive treatment of BP can reduce the risk of graft failure needs further large randomized controlled trials.

## Data Availability

The original contributions presented in the study are included in the article/**Supplementary Material**, further inquiries can be directed to the corresponding author/s.
