# Supplementary material for: Uncontrolled hypertension is associated with increased risk of graft failure in kidney transplant recipients: a nationwide population-based study
Source: Front Cardiovasc Med. 2023 Jul 14;10:1185001. doi: 10.3389/fcvm.2023.1185001 (PMC10379652; doi:10.3389/fcvm.2023.1185001)
Supplement: Supplementary file 1 [file Datasheet1.docx]

Supplementary Material

Uncontrolled hypertension is associated with increased risk of graft failure in kidney transplant recipients: A nationwide population-based study

**Supplemental Tables**

| Items | ICD-10-CM codes ^a^ | Definition |
| --- | --- | --- |
| Smoking |  |  |
| Never |  | Never-smoker or total lifetime smoking history <5 packs (100 cigarettes) |
| Former |  | Total lifetime smoking history ≥5 packs (100 cigarettes) and quit smoking |
| Current |  | Total lifetime smoking history ≥5 packs (100 cigarettes) and currently smoking |
| Alcohol consumption |  |  |
| None |  | Zero daily alcohol intake |
| Moderate |  | < 30 g of alcohol/day |
| Heavy |  | ≥ 30 g of alcohol/day |
| Regular exercise |  | Intense physical activity for at least 20 min/day for > 3 days, or moderate physical activity for at least 30 min/d for > 5 days during the previous week |
| Low income |  | Income lower <25% or the receipt of Medical Aid benefits |
| Diabetes mellitus | E11−14 | A history of glucose-lowering drug use, or recorded fasting serum glucose level ≥ 126 mg/dl in the health examination data |
| Cardiovascular disease | I21−22, I63−64 | A history of myocardial infarction or ischemic stroke. Myocardial infarction was defined as ICD-10-CM code I21 or I22 during hospitalization. Ischemic stroke was defined as ICD-10-CM code I63 or I64 during hospitalization with claims for brain magnetic resonance imaging or brain computerized tomography. |
| Dyslipidemia | E78 | A history of lipid-lowering drug use, or a total serum cholesterol level ≥ 240 mg/dl in the health examination data |

**Supplemental Table S1. Definitions and diagnostic codes**

^a^ ICD-10-CM, International Classification of Diseases, 10th Revision, Clinical Modification

**Supplementary Table S2. Subgroup analysis of HRs for death-censored graft failure according to hypertension grades stratified by age, sex, smoking, and diabetes mellitus**

| Group | Subgroup | Adjusted HR (95% CI) ^a^ | | | | | *P* for interaction |
| --- | --- | --- | --- | --- | --- | --- | --- |
|  |  | Normal BP | Elevated BP | Incident HTN without medications | Controlled HTN | Uncontrolled HTN |  |
| Age, years | 20–39 | 1 (reference) | 1.374(0.688–2.742) | 2.097(1.367–3.217) | 1.574(0.993–2.496) | 3.087(2.071–4.603) | 0.195 |
|  | 40–64 | 1 (reference) | 1.148(0.828–1.592) | 1.439(1.166–1.775) | 1.855(1.516–2.269) | 2.486(2.061–2.997) |  |
|  | ≥ 65 | 1 (reference) | 1.881(0.505–7.005) | 3.531(1.241–10.046) | 3.775(1.339–10.642) | 4.556(1.67–12.426) |  |
| Sex | Male | 1 (reference) | 0.997(0.662–1.500) | 1.426(1.104–1.843) | 1.612(1.256–2.069) | 2.255(1.788–2.844) | 0.477 |
|  | Female | 1 (reference) | 1.400(0.938–2.090) | 1.752(1.342–2.287) | 2.162(1.662–2.812) | 3.024(2.379–3.843) |  |
| Smoking | Never | 1 (reference) | 1.380(0.985–1.935) | 1.711(1.365–2.145) | 1.948(1.557–2.438) | 3.007(2.449–3.692) | 0.003 |
|  | Former | 1 (reference) | 0.741(0.389–1.413) | 1.430(0.984–2.078) | 1.391(0.969–1.996) | 1.612(1.151–2.258) |  |
|  | Current | 1 (reference) | 1.103(0.404–3.013) | 1.260(0.689–2.305) | 2.459(1.404–4.306) | 3.107(1.837–5.254) |  |
| Diabetes | No | 1 (reference) | 1.279(0.918–1.784) | 1.722(1.384–2.142) | 1.997(1.604–2.486) | 3.026(2.479–3.693) | 0.041 |
|  | Yes | 1 (reference) | 0.988(0.562–1.737) | 1.299(0.922–1.829) | 1.510(1.096–2.080) | 1.831(1.354–2.476) |  |

^a^ adjusted for age, sex, low income, smoking, alcohol consumption, regular exercise, obesity, and history of diabetes and dyslipidemia.

Abbreviations: BP, blood pressure; HTN, hypertension; HR, hazard ratio; CI, confidential interval.

**Supplementary Table S3. Subgroup analysis of HRs for death-censored graft failure according to SBP grades stratified by age, sex, smoking, and diabetes mellitus**

| Group | Subgroup | Adjusted HR (95% CI) ^a^ | | | | | *P* for interaction |
| --- | --- | --- | --- | --- | --- | --- | --- |
|  |  | < 100 mmHg | 100–119 mmHg | 120–129 mmHg | 130–139 mmHg | ≥ 140 mmHg |  |
| Age, years | 20–39 | 0.603(0.189–1.919) | 1 (reference) | 1.401(1.002–1.958) | 1.958(1.419–2.701) | 3.170(2.246–4.473) | 0.061 |
|  | 40–64 | 0.893(0.608–1.312) | 1 (reference) | 1.112(0.959–1.289) | 1.465(1.274–1.686) | 1.965(1.686–2.290) |  |
|  | ≥ 65 | 0.409(0.055–3.022) | 1 (reference) | 1.501(0.909–2.478) | 1.233(0.743–2.047) | 1.619(0.993–2.640) |  |
| Sex | Male | 1.051(0.634–1.742) | 1 (reference) | 1.156(0.976–1.369) | 1.516(1.292–1.778) | 2.068(1.742–2.455) | 0.770 |
|  | Female | 0.685(0.412–1.141) | 1 (reference) | 1.222(0.995–1.499) | 1.507(1.233–1.841) | 2.039(1.645–2.527) |  |
| Smoking | Never | 0.818(0.530–1.262) | 1 (reference) | 1.197(1.015–1.412) | 1.678(1.437–1.958) | 2.146(1.812–2.540) | 0.007 |
|  | Former | 1.062(0.494–2.282) | 1 (reference) | 1.215(0.942–1.567) | 1.364(1.062–1.751) | 1.610(1.223–2.119) |  |
|  | Current | 0.618(0.193–1.978) | 1 (reference) | 0.983(0.668–1.444) | 1.000(0.676–1.480) | 2.675(1.860–3.846) |  |
| Diabetes | No | 0.818(0.525–1.273) | 1 (reference) | 1.204(1.027–1.412) | 1.628(1.397–1.896) | 2.377(2.014–2.805) | 0.037 |
|  | Yes | 0.850(0.461–1.568) | 1 (reference) | 1.133(0.903–1.421) | 1.299(1.047–1.611) | 1.585(1.265–1.987) |  |

^a^ Adjusted for adjusted for age, sex, low income, smoking, alcohol consumption, regular exercise, obesity, and history of diabetes and dyslipidemia, use of antihypertensive medications.

Abbreviations: HTN, hypertension; HR, hazard ratio; CI, confidential interval; SBP, systolic blood pressure.

**Supplementary Table S4. Subgroup analysis of HRs for death-censored graft failure according to DBP grades stratified by age, sex, smoking, and diabetes mellitus**

| Group | Subgroup | Adjusted HR (95% CI) ^a^ | | | | | *P* for interaction |
| --- | --- | --- | --- | --- | --- | --- | --- |
|  |  | < 70 mmHg | 70–79 mmHg | 80–89 mmHg | 90–99 mmHg | ≥ 100 mmHg |  |
| Age, years | 20–39 | 0.676(0.419–1.092) | 1 (reference) | 1.477(1.120–1.947) | 2.248(1.540–3.280) | 2.760(1.737–4.386) | 0.015 |
|  | 40–64 | 0.991(0.842–1.167) | 1 (reference) | 1.170(1.034–1.323) | 1.208(1.009–1.446) | 1.971(1.538–2.526) |  |
|  | ≥ 65 | 1.012(0.635–1.613) | 1 (reference) | 1.428(0.976–2.091) | 1.327(0.771–2.282) | 0.819(0.255–2.632) |  |
| Sex | Male | 1.001(0.823–1.217) | 1 (reference) | 1.189(1.038–1.363) | 1.320(1.085–1.605) | 2.056(1.593–2.654) | 0.753 |
|  | Female | 0.924(0.741–1.151) | 1 (reference) | 1.308(1.097–1.559) | 1.369(1.060–1.767) | 1.889(1.275–2.801) |  |
| Smoking | Never | 0.958(0.799–1.148) | 1 (reference) | 1.360(1.187–1.557) | 1.424(1.172–1.730) | 2.090(1.576–2.772) | 0.003 |
|  | Former | 0.995(0.743–1.334) | 1 (reference) | 1.109(0.899–1.368) | 1.004(0.727–1.388) | 1.301(0.817–2.073) |  |
|  | Current | 0.956(0.604–1.513) | 1 (reference) | 0.879(0.629–1.227) | 1.751(1.138–2.694) | 3.052(1.901–4.902) |  |
| Diabetes | No | 0.807(0.665–0.979) | 1 (reference) | 1.286(1.128–1.466) | 1.361(1.127–1.643) | 2.346(1.833–3.003) | 0.001 |
|  | Yes | 1.235(0.985–1.550) | 1 (reference) | 1.125(0.932–1.359) | 1.295(0.983–1.706) | 1.368(0.887–2.108) |  |

^a^ Adjusted for adjusted for age, sex, low income, smoking, alcohol consumption, regular exercise, obesity, and history of diabetes and dyslipidemia, use of antihypertensive medications.

Abbreviations: HTN, hypertension; HR, hazard ratio; CI, confidential interval; DBP, diastolic blood pressure.

**Supplementary Table S5. Subgroup analysis of HRs for death-censored graft failure according to pulse pressure grades stratified by age, sex, smoking, and diabetes mellitus**

| Group | Subgroup | Adjusted HR (95% CI) ^a^ | | | | | *P* for interaction |
| --- | --- | --- | --- | --- | --- | --- | --- |
|  |  | < 40 mmHg | 40–49 mmHg | 50–59 mmHg | 60–69 mmHg | ≥ 70 mmHg |  |
| Age, years | 20–39 | 0.884(0.611–1.280) | 1 (reference) | 1.487(1.141–1.938) | 1.559(1.043–2.330) | 2.247(1.046–4.827) | 0.384 |
|  | 40–64 | 0.806(0.681–0.953) | 1 (reference) | 1.344(1.190–1.519) | 1.515(1.273–1.802) | 2.485(1.978–3.123) |  |
|  | ≥ 65 | 0.479(0.204–1.124) | 1 (reference) | 0.859(0.571–1.290) | 0.883(0.560–1.393) | 1.400(0.856–2.290) |  |
| Sex | Male | 0.796(0.652–0.972) | 1 (reference) | 1.349(1.179–1.544) | 1.438(1.193–1.735) | 2.416(1.881–3.102) | 0.960 |
|  | Female | 0.808(0.643–1.014) | 1 (reference) | 1.293(1.084–1.543) | 1.449(1.131–1.856) | 2.109(1.536–2.896) |  |
| Smoking | Never | 0.822(0.682–0.991) | 1 (reference) | 1.363(1.191–1.559) | 1.481(1.226–1.789) | 2.298(1.801–2.932) | 0.924 |
|  | Former | 0.849(0.629–1.145) | 1 (reference) | 1.309(1.057–1.621) | 1.388(1.036–1.861) | 2.122(1.428–3.152) |  |
|  | Current | 0.608(0.378–0.978) | 1 (reference) | 1.196(0.873–1.640) | 1.371(0.879–2.138) | 2.915(1.592–5.336) |  |
| Diabetes | No | 0.800(0.669–0.955) | 1 (reference) | 1.368(1.203–1.556) | 1.532(1.267–1.853) | 2.106(1.574–2.819) | 0.661 |
|  | Yes | 0.807(0.611–1.067) | 1 (reference) | 1.244(1.025–1.509) | 1.306(1.023–1.668) | 2.393(1.816–3.153) |  |

^a^ Adjusted for adjusted for age, sex, low income, smoking, alcohol consumption, regular exercise, obesity, and history of diabetes and dyslipidemia, use of antihypertensive medications.

Abbreviations: HTN, hypertension; HR, hazard ratio; CI, confidential interval.

**Supplementary Table S6. Subgroup analysis of HRs for death-censored graft failure according to hypertension categories and blood pressure stratified by duration from kidney transplantation to blood pressure measurement**

| Group | <5 years ^a^ | ≥5years ^b^ |  |
| --- | --- | --- | --- |
|  | Adjusted HR (95% CI) ^c^ | | *P* for interaction |
| Hypertension |  |  |  |
| No | 1 (reference) | 1 (reference) | 0.496 |
| Yes | 1.813(1.449,2.269) | 1.644(1.38,1.959) |  |
| Hypertension categories |  |  |  |
| Normal BP | 1 (reference) | 1 (reference) | 0.589 |
| Elevated BP | 0.986(0.604,1.61) | 1.334(0.937,1.899) |  |
| Incident HTN without medications | 1.368(1.016,1.843) | 1.518(1.201,1.918) |  |
| Controlled HTN | 1.661(1.249,2.209) | 1.543(1.224,1.946) |  |
| Uncontrolled HTN | 2.172(1.67,2.825) | 2.121(1.713,2.625) |  |
| SBP, mmHg |  |  |  |
| < 100 | 1.01(0.614,1.663) | 0.645(0.383,1.085) | 0.630 |
| 100–119 | 1 (reference) | 1 (reference) |  |
| 120–129 | 1.144(0.929,1.409) | 1.151(0.974,1.36) |  |
| 130–139 | 1.386(1.139,1.687) | 1.42(1.209,1.668) |  |
| ≥ 140 | 1.677(1.353,2.078) | 1.868(1.576,2.213) |  |
| DBP, mmHg |  |  |  |
| < 70 | 1.102(0.879,1.381) | 0.847(0.699,1.026) | 0.455 |
| 70–79 | 1 (reference) | 1 (reference) |  |
| 80–89 | 1.275(1.072,1.518) | 1.143(0.997,1.311) |  |
| 90–99 | 1.243(0.966,1.599) | 1.234(1.013,1.503) |  |
| ≥ 100 | 1.845(1.316,2.588) | 1.86(1.412,2.451) |  |
| Pulse pressure, mmHg |  |  |  |
| < 40 | 0.835(0.658,1.058) | 0.761(0.627,0.923) | 0.873 |
| 40–49 | 1 (reference) | 1 (reference) |  |
| 50–59 | 1.265(1.066,1.5) | 1.166(1.016,1.339) |  |
| 60–69 | 1.225(0.966,1.554) | 1.266(1.045,1.534) |  |
| ≥ 70 |  |  |  |

^a^ <5 years from kidney transplantation to blood pressure measurement.

^b^ ≥5 years from kidney transplantation to blood pressure measurement.

^c^ Adjusted for adjusted for age, sex, low income, smoking, alcohol consumption, regular exercise, obesity, and history of diabetes and dyslipidemia, use of antihypertensive medications.

Abbreviations: HTN, hypertension; HR, hazard ratio; CI, confidential interval.
